# Anemia and Risk Factors in HAART Naïve and HAART Experienced HIV Positive Persons in South West Ethiopia: A Comparative Study

**DOI:** 10.1371/journal.pone.0072202

**Published:** 2013-08-16

**Authors:** Lealem Gedefaw, Tilahun Yemane, Zewdineh Sahlemariam, Daniel Yilma

**Affiliations:** 1 Department of Medical Laboratory Science and Pathology, College of Public Health and Medical Sciences, Jimma University, Ethiopia; 2 Department of Internal Medicine, College of Public Health and Medical Sciences, Jimma University, Ethiopia; The George Washington University Medical Center, United States of America

## Abstract

**Background:**

Human immunodeficiency virus (HIV) infection and its treatment cause a range of hematological abnormalities. Anemia is one of the commonly observed hematologic manifestations in HIV positive persons and it has multifactorial origin.

**Objective:**

We aimed to determine the prevalence and risk factors of anemia in highly active antiretroviral therapy (HAART) naïve and HAART experienced HIV positive persons.

**Methods:**

A facility-based comparative cross sectional study was conducted in Jimma University Specialized Hospital from February 1 to March 30, 2012. A total of 234 HIV positive persons, 117 HAART naïve and 117 HAART experienced, were enrolled in this study. Blood and stool specimens were collected from each participant. Blood specimens were examined for complete blood count, CD4 count and blood film for malaria hemoparasite; whereas stool specimens were checked for ova of intestinal parasites. Socio-demographic characteristics and clinical data of the participants were collected using pre-tested questionnaire. Statistical analysis of the data (Chi-square, student’s t-test, logistic regression) was done using SPSS V-16.

**Results:**

The overall prevalence of anemia was 23.1%. The prevalence of anemia in HAART naïve and HAART experienced persons was 29.9% and 16.2%, respectively (P = 0.014). Presence of opportunistic infections (P = 0.004, 95% CI = 1.69–15.46), CD4 count <200 cells/µl (P = 0.001, 95% CI = 2.57–36.89) and rural residence (P = 0.03, 95% CI = 1.12–10.39) were found to be predictors of anemia for HAART naïve participants. On the other hand, HAART regimen (ZDV/3TC/NVP) (P = 0.019, 95% CI = 0.01–1.24) and the duration of HAART (P = 0.007, 95% CI = 0.003–0.40.24) were found to be predictors of anemia for HAART experienced groups.

**Conclusion:**

The prevalence of anemia in HAART naïve persons was higher than HAART experienced persons. Risk factors for anemia in HAART naïve and HAART experienced HIV positive persons were different. Hence, there is a need for longitudinal study to further explore the causes of HIV associated anemia and the pattern of hemoglobin changes with initiation of HAART.

## Background

Hematological complications have been documented to be the second most common causes of morbidity and mortality in HIV positive persons [1]. Anemia is one of the most commonly observed hematological abnormalities and an independent prognostic marker of HIV disease progression in people living with HIV [2], [3], [4]. The prevalence of anemia in HIV positive people has been estimated to be 63% to 95% in different study settings [4].

The cause of anemia in HIV positive people is multifactorial in origin. Infiltrations of the bone marrow by neoplasm or infection, use of myelosuppressive medications, decrease production of endogenous erythropoietin, hemolysis that may result from RBC auto antibodies and HIV infection itself are some of the causes of anemia in HIV positive people [5], [6]. The risk factors of HIV associated anemia in developing countries might differ from developed countries due to high prevalence of malnutrition, tuberculosis and helminthic infections. But there are only limited studies that have addressed the risk factors of anemia in HIV positive persons in resource-limited setting.

Anemia has negative consequences on the health and quality of life of HIV positive individuals [7]. Identifying the risk factors for anemia in this population might help to develop strategies to reduce its incidence. Therefore, the aim of this study was to assess the prevalence and risk factors of anemia among HAART naïve and HAART experienced HIV positive persons.

## Materials and Methods

A facility based comparative cross sectional study was conducted in Jimma university specialized hospital (JUSH) from February 1 to March 30, 2012. A total of 234 HIV positive participants (117 HAART naive and 117 HAART experienced) who presented to JUSH Antiretroviral (ART) clinic during the study period were included consecutively. The sample size was calculated by using statistical formula for the two population proportion of the same sample size. HIV positive participants who were on follow up during the study period and gave informed consent were included. However pregnant women, HIV positive participants who were on treatment for anemia in the last 3 months, taking nutrient supplementation for the last one year and HAART experienced persons who took HAART for less than 3 months were excluded.

Participant’s demographic and clinical information was collected by trained clinical nurses using a pre-tested questionnaire. A volume of 3–5 ml venous blood in EDTA vacutainer tube was collected by experienced laboratory technicians from each participant and sent to the hematology laboratory. The blood samples were used for complete blood count, CD4 count determination and to examine for malaria hemoparasite. In addition, a stool specimen was collected from each participant using a standard stool cup and sent to the medical parasitology laboratory.

Hematological parameters: hemoglobin(Hgb), hematocrit(Hct), Mean cell volume(MCV), Mean cell hemoglobin(MCH), Mean cell hemoglobin concentration(MCHC), red blood cell count(RBC), and red cell distribution width(RDW) were determined using the automated blood analyzer CELL DYNE 1800 (Abott Laboratories Diagnostics Division) and CD4 count was measured using BD FACS count machine. A world health organization Hgb threshold was used to classify individuals living at sea level as anemic. Based on this the hemoglobin threshold for children (0.50–4.99 yrs) <11(g/dl), children (5.00–11.99 yrs) <11.5 g/dl, children (12.00–14.99 yrs) <12.0 g/dl, non-pregnant women (≥15.00 yrs) <12.0 and men (≥15.00 yrs.) <13 g/dl was used to classify participants as anemic [8]. Microcytosis was defined as MCV<80 fl and hypochromia was defined as MCHC value<31 g/dl. Both thick and thin blood film was prepared and stained using Giemsa stain for malaria parasites. Stool examination was performed using direct wet mount and McMaster concentration technique.

To assure the quality of the data, standard operating procedures (SOPs) were followed during specimen collection and all other laboratory procedures. All reagents used were checked for their expiry date and prepared according to the manufacturer’s instructions. A control reagent was used for the hematology analyzer to check the accuracy and precision of the results. Training was given for the data collectors to minimize technical and observer biases. For statistical analysis simple frequencies, unpaired T-test, Chi square (×^2^) and multiple logistic regression analysis were performed using SPSS V-16.0. A p-value of <0.05 was considered as statistically significant.

Ethical clearance was obtained from Jimma University ethical review board and a letter of permission to conduct the study was obtained from JUSH clinical director office. Written informed consent was obtained directly from the study participants for adults and from the guardians of children enrolled in the study after providing information about the purpose and methods of the study. All abnormal results identified during laboratory investigations were reported to the health professionals working in JUSH ART clinic.

## Results

### Socio Demographic and Clinical Characteristics of Study Participants

A total of 234 HIV positive participants, *117 HAART naïve and 117 HAART experienced,* were enrolled in this study. Seventy nine (54.5%) of HAART naïve, and 66 (45.5%) of HAART experienced participants were females (Table-1). The mean (±SD) age of the study participants was 32.09±12.11 year. HAART experienced HIV positive participants were older (36.10±11.09 years, P = 0.001) than their HAART- naïve counterparts (28.08±11.81 years). There was no significant difference between HAART naïve and HAART experienced study participants with regard to the mean CD4 count (p = 0.855) (Table-2).

**Table 1:** Socio-demographic and clinical variables of HIV positive participants, South West Ethiopia, 2012.

Variables		HAART status			
		On HAART N0 (%)	HAART naïve N0 (%)	Total N0 (%)	p- value
Sex	Male	51 (57.3)	38 (42.7)	89 (38)	*0.640*
	Female	66 (45.5)	79 (54.5)	145 (62)	
Age	0–15	4 (20)	16 (80)	20 (8.5)	*0.001*
	15–30	36 (39.6)	55 (60.4)	91 (38.9)	
	30–45	55 (58.5)	39 (41.5)	94 (40.2)	
	45–60	18 (72)	7 (28)	25 (10.7)	
	> = 60	4 (100)	0 (0.0)	4 (1.7)	
Residence	Urban	103 (51.5)	97 (48.5)	200 (85.5)	*0.266*
	Rural	14 (41.2)	20 (58.8)	34 (14.5)	
Income	Low	64 (60.4)	42 (39.6)	106 (45.3)	.*001*
	Medium	19 (29.2)	46 (70.8)	65 (27.8)	
	High	34 (54)	29 (46)	63 (26.9)	
CD4 count (Cells/µl)	<200	14 (42.4%)	19 (57.6%)	33 (14.1)	*0.389*
	200–500	67 (54.0)	57 (46.0)	124 (53.0)	
	>500	36 (46.8)	41 (53.2)	77 (32.9)	
BMI (kg/m^2^)	<18.5	32 (41)	46 (59)	78 (33.3)	0.151
	18.5–24.99	75 (54.3)	63 (45.7)	138 (59)	
	>25	10 (55.6)	8 (44.4)	18 (7.7)	
OI*	Yes	24 (54.5)	20 (45.5)	44 (18.8)	0.503
	No	93 (48.9)	97 (51.1	190 (81.2)	
Chronic illness*	Yes	5 (83.3)	1 (16.7)	6 (2.6)	0.098
	No	112 (49.1)	116 (50.9)	228 (97.4)	
Other Therap. drugs	Yes	11 (37.9)	18 (62.1)	29 (12.4)	0.165
	No	106 (51.7)	99 (48.3)	205 (87.6)	
IP*	Positive	31 (58.5)	22 (41.5)	53 (22.9)	0.193
	Negative	86 (48.3)	92 (51.7)	178 (77.1)	

OI = Opportunistic infection, IP = Intestinal parasites, BMI = Body mass index, chronic illness*(Blood pressure, Diabetes, Epilepsy).

**Table 2:** Mean values of hematological and immunologic parameters of HIV positive patients stratified by HAART status, South west Ethiopia, 2012.

Parameter	All HIV patients		P-Value	T - value (95% CI)
	On HAART (mean ±SD)	HAART naïve (mean ± SD)		
RBC	4.14±.07	4.95±.057	.001	−0.07 (−1.78–1.66)
Hgb	14.10±.21	13.48±.20	.001	2.12 (0.43–1.19)
Hct	45.55±.67	45.62±.55	.944	−8.96 (−0.98–(−0.63))
MCV	109.84±1.27	92.43±.68	.001	12.07 (14.56–20.25)
MCH	34.84±.45	27.78±.24	.001	13.83 (6.06–8.07)
MCHC	31.41±.23	32.16±2.10	.723	−0.36 (−4.92–3.42)
RDW	14.57±.15	14.92±.21	.170	−1.38 (−0.85–0.15)
CD4 count	430.63±22.75	437.21±27.85	.855	−0.18 (−77.43–64.26)

SD* = standard deviation, CI* = confidence interval, RBC = red blood cell count, Hgb = Hemoglobin, Hct = Hematocrit, MCV = mean cell volume, MCH = mean cell hemoglobin, MCHC = mean cell hemoglobin concentration, RDW = red cell distribution width. CD = cluster of differentiation.

Stool samples of 231 participants were screened. One or more intestinal parasites were detected in 53 (22.9%) of participants and 58.5% of these were detected from HAART experienced participants. *Ascaris lumbricoids* 28(43.1%) was the most prevalent parasite detected, followed by *Trichuris trichiura* 26 (40%), and *Hook worm* 5(7.7%). Blood films were examined to assess for malaria hemoparasites to all participants but no hemoparasites were detected. From the total 234 individuals, 44(18.8%) of participants had opportunistic infections with the highest proportion on HAART experienced participants 24(54.5%) than HAART naïve 20 (45.5%) participants. Tuberculosis (pulmonary and extra-pulmonary) accounted the highest proportion 14 (30.43%) of opportunistic infections followed by respiratory tract infections (upper and lower) 12(26.09%), and Herpes Zoster infection 8(17.39%) (Table-1).

### Prevalence of Anemia among HIV Positive Participants

Participant’s Hgb level was used to determine the prevalence of anemia. The overall prevalence of anemia in this study was 54 (23.1%). Nineteen (16.2%) of HAART experienced and 35(29.9%) of HAART naïve participants were anemic from their respective groups (p = 0.014). On the other hand, from the total anemic individuals; 1(1.9%), 14(25.9%), and 39(72.2%) had sever, moderate, and mild anemia, respectively. No significant difference was found in the degree of anemia between HAART naïve and HAART experienced study participants (P = 0.203). The type of anemia was determined using red cell indices values and macrocytosis (MCV>100 fl) was found to be more common in HAART experienced patients 85.7% (12/14) compared to HAART naïve patients 14.3% (2/14).

The Mean ± SD of Hgb in HAART experienced and HAART naïve participants was 14.10±2.28 g/dl and 13.48±2.18 g/dl, respectively. Statistically significant difference was observed between HAART naïve and HAART experienced participants with regard to the mean Hgb level (t = 2.12 [0.043–1.19]). There was also significant difference on mean value of RBC, MCV, and MCH between HAART experienced and HAART naïve participants (p<0.05).

### Factors Associated with Anemia in Participants with HIV/AIDS

Both binary and multiple logistic regression analysis were performed to identify the risk factors of anemia in HAART naïve and HAART experienced HIV positive persons. The variables; age, sex, residence, BMI, intestinal parasitosis, CD4 count, presence of opportunistic infections, HAART regimen, duration of HAART were included in the analysis. After adjusting for these factors in a multiple logistic regression analysis for both groups; presence of opportunistic infections, CD4 count <200 cells/µl and rural residence for HAART naïve participants; HAART regimen and duration of HAART for HAART experienced groups were remained independently and significantly associated with anemia (Table-3 &4).

**Table 3:** Multiple logistic regression analysis for predictors of anemia in HAART naïve HIV patients involved in the study, South west Ethiopia, 2012.

Variables		COR* (95% CI*)	P-value	AOR* (95% CI*)	p-value
**Opportunistic infection**	Yes	4.83 (1.76–13.25)	.002	5.12 (1.69–15.46)	0.004
	No	1 (ref)		1 (ref)	
**CD4 count(cells/µl)**	<200	8.33 (2.42–28.69)	.001	9.73 (2.57–36.89)	.001
	200–499	1.90 (0.70–5.14)	.209	1.69 (0.57–4.98)	0.344
	>500	1 (ref)		1 (ref)	
**Residence**	Urban	1 (ref)		1 (ref)	
	Rural	3.72 (1.38–10.05)	0.010	3.42 (1.12–10.39)	0.030

NB: COR* = Crude Odds ratio, AOR* = adjusted odds ratio, CI* = Confidence interval.

**Table 4:** Multiple logistic regression analysis for predictors of anemia in HAART experienced HIV patients involved in the study, south west Ethiopia, 2012.

Variables		COR (95% CI)	P-value	AOR (95% CI)	p-value
**HAART regimen**	D4T/3TC/NVP	0.074 (0.008–0.714)	0.024	0.11 (0.01–1.24)	0.074
	ZDV/3TC/NVP	0.148 (0.025–0.89)	0.037	0.09 (0.012–0.68)	0.019
	TDF/3TC/NVP	0.67 (0.097–4.58)	0.68	0.98 (0.12–8.99)	0.987
	TDF/3TC/EFV	0.76 (0.198–2.93)	0.69	0.301 (0.058–1.57)	0.154
	D4T/3TC/EFV	0.286 (0.027–3.01)	0.297	0.37 (0.03–4.82)	0.446
	ZDV/3TC/EFV	1 (ref)		1 (ref)	
**Duration of HAART** **in months**	3–20	1 (ref)		1 (ref)	
	20–36	00.242 (0.067–0.881)	0.031	.023 (0.038–0.789)	.023
	36–60	0.043 (0.005–0.36)	0.004	.007 (0.003–0.404)	.007
	>60	0.327 (0.077–1.394)	0.131	.215 (0.053–1.932)	.215

NB: ZDV = Zidovudine, d4T = Stavudine, 3TC = Lamivudine, NVP = Neverapine, TDF = Tenofovir, EFV = Efavirenz.

## Discussions

The prevalence of anemia in HAART naïve HIV positive participants was higher than HAART experienced participants. Opportunistic infection, CD4 count <200 cells/µl and rural residence in HAART naïve patients; HAART regimen and duration of HAART in HAART experienced patients were identified as risk factors for anemia.

The overall prevalence of anemia (23.1%) was high as compared to studies conducted in Iran (2009) and Uganda (2008) which reported 10.3% and 18.9%, respectively [2], [9]. However, the finding of this study is lower than the studies done in India (2009), Nigeria (2009) and USA (2007) which showed prevalence of 65.5%, 60.61%, and 36%, respectively [10], [11], [12]. This difference might be due to socio-economic, geographic and methodological variations.

The prevalence of anemia was higher and the mean Hgb level was lower in HAART naïve participants compared to HAART experienced study participants. This finding is supported by Owiredu WKBA *etal* [1], Richard D. *et al* [13], and Asgeir J. *et al* [14].This may indirectly indicate the effectiveness of HAART in reducing HIV associated anemia by reducing the occurrence of opportunistic infections, anemia of chronic disease, and by improving the nutritional status of the patients. The overall improvement in Hgb concentration on HAART experienced patients in this study may also additionally confirm the effectiveness of HAART in improving the anemia in HIV positive participants. On the other hand, macrocytosis was more common in HAART experienced 12(85.7%) than HAART naïve participants 2(14.3%). The elevated MCV observed in HAART experienced patients in this study could therefore be attributed to the effect of zidovudine (ZDV) as most of them were on a combination therapy of ZDV and lamivudine (3TC).

The prevalence of anemia was found to be higher among children (<15 years age) and rural residents. This higher prevalence of anemia in children could be explained by the accelerated growth and consequent increased requirement for iron during their development in addition to the disease state. However, the high prevalence in rural residents might be due to the fact that those participants residing in rural areas might not have adequate information about nutrition and other factors that could cause anemia.

Opportunistic infections, CD4 count <200 cells/µl and rural residence were identified as the predictors of anemia in HAART naïve participants. Patients with opportunistic infections had 5.12 times more risk of developing anemia compared to those without opportunistic infections (P = 0.004, 95% CI = 1.69–15.46). The chance of developing anemia in patients with CD4 count <200 cells/µl was 9.73 times more than those with CD4 count of ≥500 cells/µl (p = 0.001, 95% CI = 2.57–36.89). This may indicate that the likelihood of anemia increases with immunologic deterioration and with the advancement of HIV-related disease. A study by Sara Jam et al. also showed that CD4 count <200 cells/µL was independently associated with the development of anemia (2). On the other hand, HAART regimen and the duration of HAART were found to be the predictors of anemia in HAART experienced HIV positive participants. This might be due to ZDV toxicity as most patients were in ZDV based HAART regimen.

In conclusion, this study has shown that HAART initiation might have important role in reducing anemia in HIV positive persons. However, HAART experienced persons also had anemia with risk factors different from HAART naïve HIV positive persons. Therefore, further longitudinal studies with long term follow-up are needed to explore more on the causes of anemia and the pattern of hemoglobin changes with HAART initiation in HIV positive persons in resource limited settings.
